# TNFR2 induced priming of the inflammasome leads to a RIPK1-dependent cell death in the absence of XIAP

**DOI:** 10.1038/s41419-019-1938-x

**Published:** 2019-09-20

**Authors:** Janin Knop, Lisanne M. Spilgies, Stefanie Rufli, Ramona Reinhart, Lazaros Vasilikos, Monica Yabal, Erika Owsley, Philipp J. Jost, Rebecca A. Marsh, Harald Wajant, Mark D. Robinson, Thomas Kaufmann, W. Wei-Lynn Wong

**Affiliations:** 1 Institute of Experimental Immunology, University of Zürich, Zürich, Switzerland; 2 Institute of Pharmacology, University of Bern, Bern, Switzerland; 3 III. Medizinische Klink, Klinikum rechts der Isar, Technische Universität München, Munich, Germany; 4 UC Department of Pediatrics, Cincinnati Children’s Hospital, Cincinnati, USA; 5 Division of Molecular Internal Medicine, Department of Internal Medicine II, University Hospital Würzburg, Würzburg, Germany; 6 Institute of Molecular Life Sciences and SIB Swiss Institute of Bioinformatics, University of Zürich, Zürich, Switzerland

**Keywords:** Cell death and immune response, Inflammation

## Abstract

The pediatric immune deficiency X-linked proliferative disease-2 (XLP-2) is a unique disease, with patients presenting with either hemophagocytic lymphohistiocytosis (HLH) or intestinal bowel disease (IBD). Interestingly, XLP-2 patients display high levels of IL-18 in the serum even while in stable condition, presumably through spontaneous inflammasome activation. Recent data suggests that LPS stimulation can trigger inflammasome activation through a TNFR2/TNF/TNFR1 mediated loop in *xiap*^*−/−*^ macrophages. Yet, the direct role TNFR2-specific activation plays in the absence of XIAP is unknown. We found TNFR2-specific activation leads to cell death in *xiap*^*−/−*^ myeloid cells, particularly in the absence of the RING domain. RIPK1 kinase activity downstream of TNFR2 resulted in a TNF/TNFR1 cell death, independent of necroptosis. TNFR2-specific activation leads to a similar inflammatory NF-kB driven transcriptional profile as TNFR1 activation with the exception of upregulation of NLRP3 and caspase-11. Activation and upregulation of the canonical inflammasome upon loss of XIAP was mediated by RIPK1 kinase activity and ROS production. While both the inhibition of RIPK1 kinase activity and ROS production reduced cell death, as well as release of IL-1β, the release of IL-18 was not reduced to basal levels. This study supports targeting TNFR2 specifically to reduce IL-18 release in XLP-2 patients and to reduce priming of the inflammasome components.

## Introduction

Full length tumor necrosis factor (TNF) is a membrane bound protein, where the extracellular domain can be cleaved by TNF converting enzyme (TACE) to release a soluble form^1^. Soluble and membrane TNF can bind and activate TNF receptor 1 (TNFR1) while only the membrane bound form triggers TNFR2 activation^2^. The outcome of TNF/TNFR1 signaling can range from production of other cytokines, proliferation, survival and differentiation. While activation of TNFR1 does not normally lead to cell death, the capacity for TNFR1 to induce apoptosis or necroptosis is swayed by the ubiquitylation and phosphorylation of RIPK1 and the activation of pro-survival signals mediated by NF-κB and MAPK pathways^3–5^. Activation of complex II is blocked by cFLIP, which is transcribed upon TNF/TNFR1 activation, preventing caspase-8 activity. In the absence or inhibition of caspase-8 activity, necroptosis ensues through RIPK1 kinase activity, RIPK3 and MLKL necrosome activity^6,7^. Phosphorylation of MLKL causes a conformational change, allowing for pore formation and the release of intracellular components, as well as damage-associated molecular patterns (DAMPs)^8^.

The expression of TNFR2 on cells of the immune system and endothelial cells is highly regulated. Upon binding of membrane-bound TNF to TNFR2^9^ a complex is formed that consists of TRAF2, cIAP1, cIAP2 and TRAF3^10^. The activation leads to the degradation of cIAP1 and TRAF2, and signals through the non-canonical NF-κB pathway^11^. Due to the absence of a death domain, TNFR2 is considered to be mainly involved in survival and maturation of immune cells. Yet, previous data suggest that in some tumor cell lines, TNFR2 can regulate cell death by the loss of cIAP1 and TRAF2, causing production of TNF and thereby TNFR1 activation^12^. In addition, immortalized macrophages died in response to TNFR2 via necroptosis when caspase activity was inhibited^13,14^.

In response to toll-like receptor (TLR) activation, XIAP deficient myeloid cells undergo TNF-dependent cell death, resulting in the activation of the inflammasome through RIPK3/caspase-8/caspase-1^15–17^. The stimulation of TLRs upregulate inflammasome components such as NLRP3, thus priming the cell^18,19^. A secondary stimulus is required to activate the inflammasome in vitro. When caspase-1 is cleaved and activated, downstream targets IL-1β and gasdermin D are cleaved. Cleaved gasdermin D forms a pore like structure that facilitates the release of cleaved IL-1β, IL-18 and the osmotic lysis of the cell^20,21^. In response to TLR stimulation, *xiap*^*−/−*^ macrophages showed increased inflammasome activation compared to wildtype through the activation of TNFR2 and subsequent degradation of cIAP1/TRAF2^22^.

We sought to understand what function TNFR2 played in the absence of XIAP when normally it would not be expected to cause cell death in primary macrophages. Specific TNFR2 stimulation alone in XIAP deficient macrophages resulted in cell death due to the lack of E3 ligase activity. RIPK1 kinase activity was required for both the TNF production from TNFR2 stimulation, as well as the TNFR1 mediated cell death. Surprisingly, neither necroptosis nor apoptosis occurred in comparison to previously published results^13,22^. Instead, we found that TNFR2 activation acts as a signal 1 for priming the inflammasome in primary macrophages independent of genotype, and the combination of XIAP loss and TNFR1 activation plays a role as signal 2 for activation. Interestingly, cell death was blocked by RIPK1 kinase inhibitor as well as reactive oxygen species (ROS) scavengers, and while many of the pro-inflammatory cytokines returned to baseline, IL-18 required genetic mutation of RIPK1 kinase to reduce to baseline levels. Thus, our work separates a key cytokine implicated in the etiology of XIAP deficient patients (X linked lymphoproliferative disease 2; XLP-2) from cell death. Taken together, we discovered a novel role of XIAP to inhibit TNFR2 induced inflammatory pyroptosis, adding additional complexity to treat XLP-2 patients.

## Results

### Loss of XIAP RING domain sensitizes myeloid cells to TNFR2 induced cell death

**Fig. 1:**
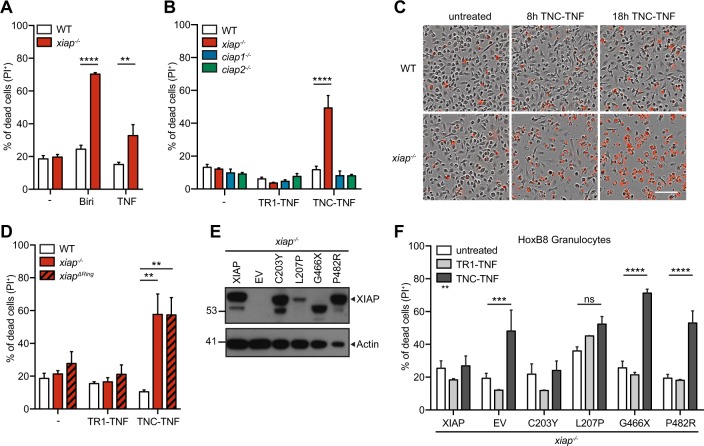
Loss of XIAP results in TNFR2-mediated cell death in myeloid cells. **a, b, d, f** Cell death was measured by the uptake of propidium iodide (PI) and analyzed by flow cytometry. **a** BMDMs from wildtype (WT) and *xiap*^*−/*^^−^ were stimulated with Birinapant (Biri, 500 nM) and recombinant mouse TNF (100 ng/ml) overnight. **b** BMDMs from WT, *xiap*^*−/*^^−^, *ciap1*^*−/*^^−^ and *ciap2*^*−/*^^−^ mice were treated overnight with TR1-TNF (100 ng/ml) or TNC-TNF (100 ng/ml). **c** Representative phase-contrast images merged with PI positive images at 0, 8 and 18 h after TNC-TNF stimulation (3 independent experiments performed). **d** BMDMs from WT, *xiap*^*−/*^^−^ and *xiap*^*Δ−/*^^−^ mice were treated overnight with TR1-TNF or TNC-TNF. **e** Basal expression levels of XIAP in HoxB8 progenitors were assessed by western blot. Blot is a representative of three independent experiments. **f** *Xiap*^*−/−*^ HoxB8 granulocytes were transfected with WT, XIAP or XIAP mutated constructs and stimulated with TR1-TNF or TNC-TNF for 24 h. **a**, **b**, **d** Data shown are mean ± SEM including *n* = 3–5 biological replicates. Experiments were repeated at least three times independently or **f** a minimum of three independent experiments, including triplicates for each experiment. Statistical significance was calculated using (**a**, **b**, **d**) two-way ANOVA or (**f**) Student *t*-test with **p < 0.01, ***p < 0.001 and ****p < 0.0001.

Similar to others, we found recombinant mouse TNF alone caused cell death in XIAP deficient macrophages^15,23^ (Fig. 1a). To assess if specific activation of TNFR1 or TNFR2 was responsible, we used human TNF (TR1-TNF) to activate only TNFR1 and the published nonameric mouse TNF fusion protein, TNC-sc(mu)TNF80 (TNC-TNF), to specifically activate TNFR2^24^. Specificity of these ligands was confirmed by using *tnfr1*^*−/−*^*tnfr2*^*−/*−^ fibroblasts with re-introduced TNFR1 or TNFR2. Briefly, the cytoplasmic portion of human TNFR1 or TNFR2 was replaced with Fas and activation of TNFR1 or TNFR2, with the respective ligands led to cell death^25^ (Fig. S1A-B). Western blots for NF-κB signaling (activation of p-p65 or p100 cleavage) were performed on *tnfr1*^−*/*−^ and *tnfr2*^*−/−*^ macrophages to confirm specificity in macrophages (Fig. S1C). Bone marrow derived macrophages (BMDMs) from wildtype, *xiap*^*−/*−^, *ciap1*^*−/−*^ and *ciap2*^*−/−*^ mice were treated with either TR1-TNF or TNC-TNF overnight and assayed for cell death (Fig. 1b). Interestingly, *xiap*^−*/−*^ macrophages but not *ciap1*^−*/*−^ or *ciap2*^*−/*−^ macrophages were sensitive to TNFR2 induced cell death but insensitive to TNFR1 stimulation. The same was seen when we stimulated freshly isolated macrophages (CD11b^+^F4/80^+^) from *xiap*^−*/*−^ bone marrow (Fig. S1D). To determine the kinetics of the observed cell death, BMDMs were imaged using time-lapse photography for the uptake of PI. *Xiap*^−*/−*^ macrophages started to die by 8 h after TNC-TNF treatment as shown by the increase in PI positivity compared to wildtype cells (Fig. 1c and S1E). Using *xiap*^*Δ*^^*RING*^ macrophages^26^, we found the E3 ligase activity required for resistance to TNC-TNF induced cell death (Fig. 1d).

Various mutations in the coding region of XIAP have been identified, contributing to immune hyper-activation and tissue inflammation in XLP-2 patients^27^. To determine whether BIR/caspase binding or RING/E3 ligase activity in identified human XIAP mutations could be associated with TNFR2 induced cell death, we utilized the HoxB8 progenitor system with re-introduced identified patient mutations^17^. Induced HoxB8 expression retains the cells in a myeloid progenitor-like state and removal of HoxB8 expression results in granulocyte differentiation. Both wildtype and *xiap*^*−/*−^ HoxB8 progenitor cells were insensitive to TNFR2-induced cell death similar to previous reports^13^ (Fig. S1F). To assess the contribution of either the BIR or RING domain of XIAP to the observed cell death, XIAP constructs containing mutations found in XLP-2 patients were re-introduced into the HoxB8 progenitor cells and expression levels assessed by western blotting^17^ (Fig. 1e). Differentiated *xiap*^*−/−*^ granulocytes were sensitive to TNFR2 induced cell death but not to TR1-TNF, while re-introduction of XIAP into the cells reduced TNFR2 induced cell death (Fig. 1f). Intriguingly, C203Y mutation in the BIR2 domain reduced the sensitivity of HoxB8 granulocytes to TNC-TNF while G466X and P482R were unable to rescue TNFR2 induced cell death (Fig. 1f). The expression levels for L207 construct were low and subsequently, sensitivity to TNC-TNF induced death occurred but was not significant. These data suggest that activation of TNFR2 in the absence of XIAP, specifically the E3 ligase domain, results in cell death in the myeloid compartment.

### TNFR2 stimulation leads to soluble TNF production resulting in TNFR1 mediated cell death in *xiap*^*−/−*^ macrophages

**Fig. 2:**
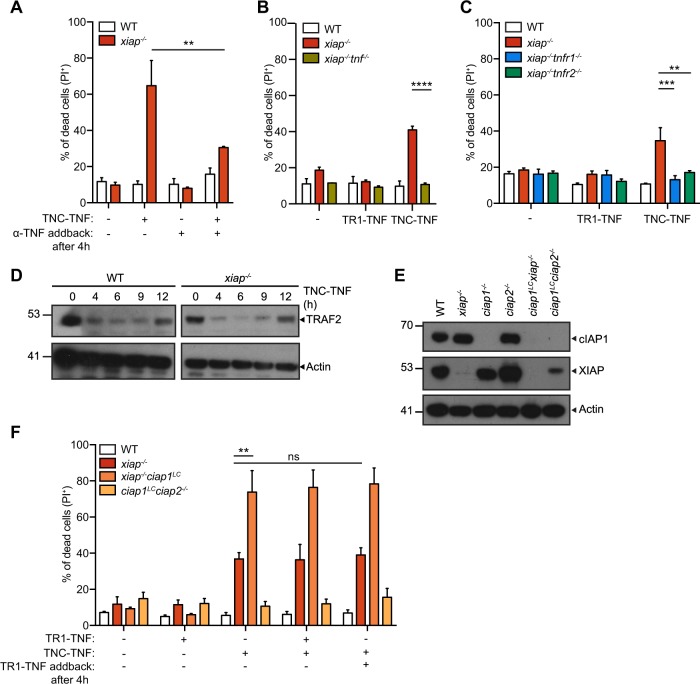
TNFR2-induced cell death in *xiap*^*−/−*^ macrophages requires soluble TNF and TNFR1 activation. **a** BMDMs from WT and *xiap*^*−/−*^ were treated with TNC-TNF and/or anti-TNFα (100 ng/ml) after 4 h of stimulation, and after 24 h cell death was measured via PI uptake by flow cytometry. **b**, **c** BMDMs from WT, *xiap*^*−/−*^, *xiap*^*−/−*^ *tnf*^*−/−*^, *xiap*^*−/−*^ *tnfr1*^*−/−*^ and x*iap*^*+*^ *nfr2*^*−/−*^ were treated with either TR1-TNF or TNC-TNF. **d** Representative western blot shows that TRAF2 degrades in both WT and *xiap*^*−/−*^ BMDMs treated with TNC-TNF. **e** Representative western blot shows loss of cIAP1 and XIAP in macrophage-specific (LC) genotypes. Blots are representative of three independent experiments. **f** WT, *xiap*^*−/−*^, *xiap*^*−/−*^*ciap1*^*LC*^ and *ciap1*^*LC*^*ciap2*^*−/−*^ BMDMs were treated with TR1-TNF, TNC-TNF, either a combination of TR1- and TNC-TNF or an initial stimulation with TNC-TNF for 4 h and subsequent TR1-TNF stimulation. Data shown are mean ± SEM including *n* = 3–5 biological replicates. Experiments were repeated at least three times independently. Statistical significance was calculated using two-way ANOVA with **p < 0.01, ***p < 0.001 and ****p < 0.0001.

Our previous data and others suggested that the inhibition or loss of XIAP, cIAP1, and cIAP2 leads to TNF production which may lead to TNFR1 mediated cell death^13,23^. To test if TNFR2 activation results in subsequent release of TNF and TNFR1 activation, we stimulated macrophages for 4 h with TNC-TNF and subsequently added anti-TNFα to neutralize any TNF being produced upon the stimulation. TNC-TNF induced cell death was reduced significantly (~30%) (Fig. 2a). In agreement, x*iap*^−*/*−^*tnf*^−*/−*^ macrophages were resistant to TNC-TNF induced cell death (Fig. 2b). Co-loss of TNFR1 or TNFR2 with XIAP deficient macrophages shows both TNFR1 and TNFR2 is required for cell death mediated by TNC-TNF (Fig. 2c). To determine if soluble TNF was produced, supernatant transfers from wildtype and *xiap*^−*/*−^ macrophages treated with TNC-TNF to TNFR1-Fas mouse fibroblast cells (TNFR1-Fas MF) showed TNFR1 mediated cell death was induced by soluble TNF produced by XIAP deficient macrophages (Fig. S2A).

To determine if the loss of cIAP1 or TRAF2 sensitized to TNFR1 mediated cell death as previously shown^22^, we assayed for degradation of TRAF2 in response to TNC-TNF over time in wildtype and *xiap*^*−/−*^ macrophages. Degradation of TRAF2 occurred by 4 h in both wildtype and *xiap*^*−/*−^ macrophages (Fig. 2d). We subsequently treated wildtype, *xiap*^*−/*−^, *xiap*^−*/*−^*ciap1*^*LysMcre*^ (*xiap*^*−/*−^*ciap1*^*LC*^) or *ciap1*^*LysMcre*^*ciap2*^*−/*−^ (*ciap1*^*LC*^*ciap2*^−*/*−^) macrophages with TR1-TNF and/or TNC-TNF. Interestingly, despite sufficient loss of cIAP1 in *ciap1*^*LC*^*ciap2*^*−/*−^ and *xiap*^−*/*−^*ciap1*^*LC*^ macrophages (Fig. 2e), neither genotypes were sensitive to TR1-TNF specific cell death. Pre-incubation with TNC-TNF for 4 h and subsequent stimulation with TR1-TNF did not sensitize wildtype cells to cell death nor did it trigger or enhance cell death in *ciap1*^*LC*^*ciap2*^*−/−*^ and *xiap*^*−/*−^*ciap1*^*LC*^ macrophages, respectively (Fig. 2f). These results suggest that TNFR2 specific activation initiates a cell death via TNF/TNFR1 in the absence of XIAP but the reduction of cIAP1/TRAF2 is not sufficient to trigger TNF/TNFR1 mediated cell death.

### TNFR2 mediated cell death in XIAP deficient macrophages is RIPK1 kinase activity-dependent but independent of downstream necroptotic machinery or apoptosis

**Fig. 3:**
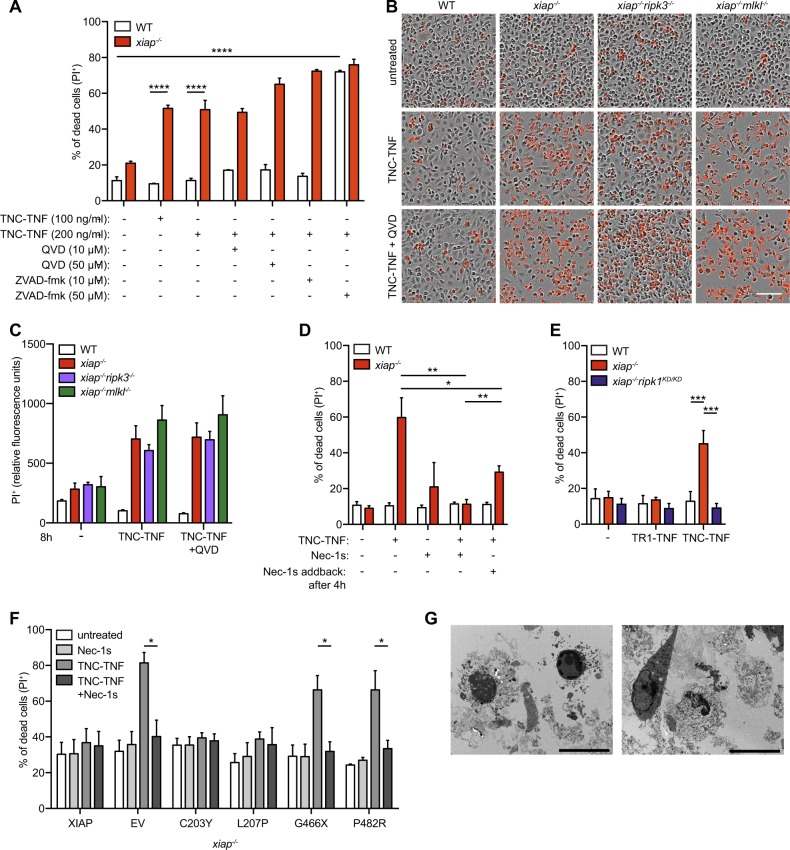
RIPK1 kinase activity mediates TNFR2-induced cell death in *xiap*^*−/−*^ macrophages, independent of apoptosis and necroptosis. **a** WT or *xiap*^*−/−*^ BMDMs were treated overnight with TNC-TNF in combination with either the caspase inhibitor QVD (10 or 50 μM) or ZVAD-fmk (10 or 50 μM). Cell death was measured via PI uptake by flow cytometry. **b** Representative phase-contrast images at 18 h after TNC-TNF and QVD (5 μM) stimulation in WT, *xiap*^*−/−*^*, xiap*^*−/−*^*ripk3*^*−/−*^ *and xiap*^*−/−*^*mlkl*^*−/−*^ BMDMs monitored for cell death by PI uptake using time-lapse photography. **c** PI^+^ BMDMs after 8 h stimulation with TNC-TNF and/or QVD using time-lapse photography. **d** BMDMs were treated with TNC-TNF in combination with the RIPK1 kinase inhibitor Necrostatin-1s (Nec-1s, 1 μM) or TNC-TNF pre-stimulation for 4 h and additional Nec-1s treatment. After 24 h cell death was measured by PI uptake and analysed by flow cytometry. **e** WT, *xiap*^*−/−*^ *or xiap*^*−/−*^*ripk1*^*KD/KD*^ BMDMs were treated with TNC-TNF and after 24 h analysed via PI uptake by flow cytometry. **f** *Xiap*^*−/−*^ HoxB8 granulocytes were transfected with WT, XIAP or XIAP mutated constructs and stimulated with TNC-TNF or in combination with Nec-1s for 24 h. **g** Transmission electron microscopy (TEM). *Xiap*^*−/−*^ BMDMs were treated for 12 h with TNC-TNF, fixed and prepared for analysis. Representative images are shown. Bars, 10 μm. **a**, **c**, **d**, **e** Data shown are mean ± SEM including *n* = 3–5 biological replicates. Experiments were repeated at least three times independently or (**f**) a minimum of three independent experiments, including triplicates for each experiment. Statistical significance was calculated using (**a**, **c**, **d**, **e**) two-way ANOVA or (**f**) Student *t*-test with *p < 0.05, **p < 0.01, ***p < 0.001 and ****p < 0.0001.

To determine the activity of caspases in TNFR2 mediated cell death, macrophages were treated with TNC-TNF and pancaspase inhibitors, QVD or ZVAD-fmk. TNFR2 induced cell death in *xiap*^*−/−*^ macrophages was slightly enhanced in the presence of QVD, suggesting cells switched from apoptosis to necroptosis, but no sensitization of wildtype cells to TNFR2 mediated cell death was seen. By contrast, use of ZVAD-fmk sensitized wildtype macrophages to TNFR2 mediated cell death as previously reported^13^ (Fig. 3a). These results suggest the type of cell death mediated in the absence of XIAP is mechanistically different to combined use of ZVAD and TNC-TNF^13^. In agreement, no caspase-3 activity upon TNC-TNF stimulation was detected (Fig. S3A)^23^. To genetically confirm if necroptosis occurs, *xiap*^*−/*−^*ripk3*^*−/*−^ and *xiap*^−*/−*^*mlkl*^*−/*−^ macrophages were treated with TNC-TNF and imaged over time (Fig. 3b and Fig. 3SB). Loss of RIPK3 or MLKL in XIAP deficient cells did not rescue macrophages from TNFR2 mediated cell death. Furthermore, co-incubation with QVD did not rescue *xiap*^*−/−*^*ripk3*^*−/−*^ or *xiap*^−*/*−^*mlkl*^*−/−*^ from TNFR2 mediated cell death, suggesting no switch from apoptosis to necroptosis or vice versa when one pathway is blocked (Fig. 3c). However, co-incubation with RIPK1 kinase inhibitor (Necrostatin-1s, Nec-1s) entirely rescued TNC-TNF induced cell death. Even after 4 h of pre-stimulation with TNC-TNF, the inhibitor significantly rescued cell death (Fig. 3d). We further confirmed our findings using macrophages from *xiap*^*−/−*^*ripk1*^*K45A/K45A*^ (*xiap*^*−/−*^*ripk1*^*KD/KD*^), a kinase inactive mutant^28^. Consistent with Necrostatin-1s, macrophages from *xiap*^−*/*−^*ripk1*^*KD/KD*^ mice were insensitive to TNC-TNF induced cell death (Fig. 3e). Similarly, HoxB8 granulocytes were resistant to TNC-TNF in the presence of Nec-1s (Fig. 3f). Using transmission electron microscopy, we identified cell membrane loss and limited membrane blebbing in *xiap*^*−/−*^ macrophages treated with TNC-TNF suggesting that the cells were dying in a lytic fashion. Taken together, TNFR2 induced cell death in the absence of XIAP is dependent on RIPK1 kinase activity in myeloid cells.

### TNFR2 acts as a signal 1 in inflammasome activation

**Fig. 4:**
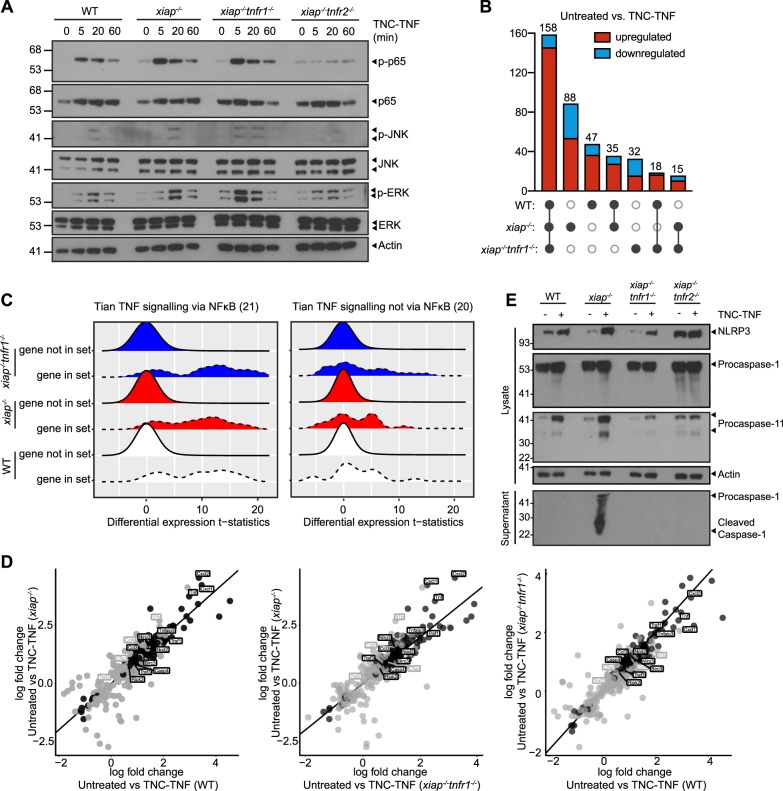
TNFR2 primes inflammasome components. **a** BMDMs were treated with TNC-TNF for the indicated time points and expression levels of proteins of the NF-κB and MAPK signaling pathway were analysed by western blotting. Blots are representative of three independent repeats. **b** Upset plot representing the detected changes in expression between untreated and TNC-TNF treated for each of the genotypes, including up- and downregulated genes. **c** Gene set analysis of Tian TNF signalling via NF-κB or not via NF-κB (number of genes in the gene set are given in parentheses). The distributions of differential expression *t*-statistics (computed by voom) are shown for the genes within the gene set and not within the gene set for each genotype. **d** Comparison of differentially regulated genes in WT, *xiap*^*−/−*^ *and xiap*^*−/−*^*tnfr1*^*−/−*^ macrophages after TNC-TNF treatment. Black dots represent genes that are differential at FDR < 0.05 in all three genotypes, and grey dots represent genes that are differential at FDR < 0.05 in two genotypes. **e** Representative western blot showing TNC-TNF treatment leads to upregulation of NLRP3, caspase-11 and pro-caspase-1, but only in *xiap*^*−/−*^ macrophages is caspase-1 cleavage detected. Blots are representative of three independent experiments.

To determine if XIAP influences downstream signaling of TNFR2, we probed for changes in NF-κB and MAPK pathways. In *xiap*^*−/−*^ macrophages, phosphorylation of p65 (p-p65) was prolonged compared to wildtype macrophages. Prolonged phosphorylation of p65 was also detected in *xiap*^*−/−*^*tnfr1*^*−/−*^ treated with TNC-TNF while *xiap*^*−/−*^*tnfr2*^*−/−*^ macrophages did not show any activation of NF-κB and MAPK pathways, further confirming the specificity of TNC-TNF for TNFR2 and that the signaling changes are independent of TNFR1 (Fig. 4a). To determine if the sustained p65 signaling altered transcription in a XIAP specific manner, we profiled wildtype, *xiap*^*−/−*^ and *xiap*^*−/−*^*tnfr1*^*−/−*^ macrophages treated with TNC-TNF for 2 h. Comparison of the untreated samples of each genotype showed the gene expression was surprisingly similar (Fig. 4b and Supp Table 1). Upon TNC-TNF stimulation, there were 88 genes uniquely regulated by the loss of XIAP, 47 in wildtype and 32 genes in *xiap*^*−/−*^*tnfr1*^*−/−*^ macrophages. Only 15 genes were differentially regulated and overlapping between *xiap*^*−/−*^ and *xiap*^*−/−*^*tnfr1*^*−/−*^ macrophages when treated with TNC-TNF, suggesting changes in this set of genes was not influenced by TNFR1.

Gene ontology analysis showed an enrichment of genes involved in inflammation and cell death processes in a similar expression pattern independent of genotype (Fig. S4A). Surprisingly, gene set enrichment analysis showed that the differentially regulated genes upon TNFR2 stimulation were alike to those associated with TNF/TNFR1, particularly genes requiring NF-kB (Fig. 4c and Supp Table 2). Genes upregulated or downregulated in LPS gene signatures and KEGG NOD-like receptor signaling were also significantly enriched in our gene sets showing the cross over of pathways linking LPS and TNF induction (Supp Table 2). These data surprisingly show that XIAP loss was a minor influence in TNFR2-induced transcriptional changes but that TNFR2 itself is a driver for inflammation in macrophages.

Consistent with others^14^, we also found, *tnfaip3* (A20) and *traf1* upregulated by TNC-TNF in our RNAseq analysis (Fig. 4d and Supp Table 1). The anti-apoptotic response of *cflar* (c-FLIP) and *birc3* (cIAP2) is consistent in *xiap*^*−/*−^ and *xiap*^*−/−*^*tnfr1*^−*/−*^ macrophages (Fig. 4d). The log fold change plots show that while *tnf, cxcl1, cxcl2, and ccl3* are differentially regulated at the RNA level in all genotypes, there is a slight increase in RNA levels in the absence of XIAP or co-loss with TNFR1 (Fig. 4d, coded in black). *Ccl4* and *il-1β* were exclusively upregulated in *xiap*^−*/−*^ macrophages (Fig. 4d, coded in gray). These data suggest that while TNFR2 directly stimulates the expression of key cytokines and chemokines, the cytokine network is further enhanced by the absence of XIAP and the stimulation of TNFR1. Interestingly, our analysis showed that in all wildtype, *xiap*^*−/−*^ and *xiap*^−*/−*^*tnfr1*^*−/*−^ macrophages, TNFR2 stimulation induced inflammasome related components such as NLRP3 and caspase-11 (*casp4)*. These results suggest that TNFR2 activation not only acts as a signal 1 for inflammasome priming but also induces the expression of cytokines and chemokines similar to TNFR1.

To confirm the involvement of the pyroptotic components in TNFR2 induced cell death, we treated WT, *xiap*^*−/−*^, *xiap*^*−/*−^*tnfr1*^−*/*−^ and *xiap*^*−/−*^*tnfr2*^*−/−*^ macrophages with TNC-TNF overnight and assayed the supernatant as well as the lysate for inflammasome activation. We found that indeed upon TNC-TNF stimulation, NLRP3 and caspase-11 were upregulated (Fig. 4e). However, the activation of caspase-1 and cleavage of gasdermin D was only observed in XIAP deficient cells. The increase in gasdermin D, caspase-1 and NLRP3 by TNC-TNF was independent of TNFR1. These results suggest TNFR2 activation alone can act as a signal 1 in inflammasome activation.

### Pro-inflammatory cytokines induced by TNFR2 are dependent on TNF and RIPK1 kinase activity

**Fig. 5:**
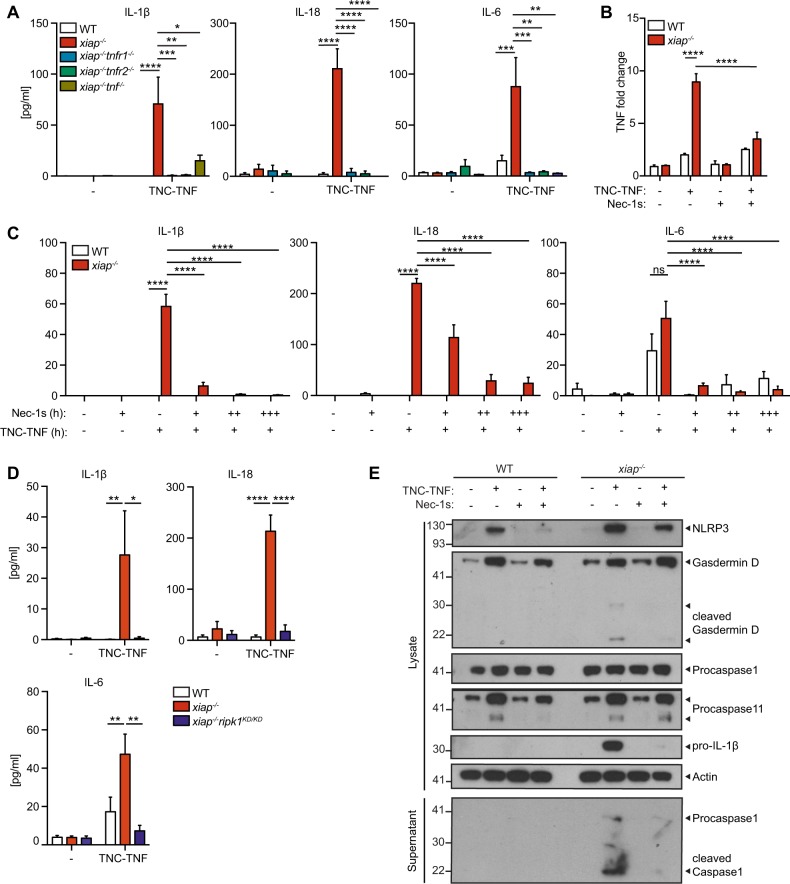
RIPK1 kinase activity regulates cytokine production and inflammasome activation in *xiap*^*−/−*^ macrophages. **a** WT, *xiap*^*−/−*^, *xiap*^*−/−*^*tnf*^*−/−*^, *xiap*^*−/−*^*tnfr1*^*−/−*^ and *xiap*^*−/−*^*tnfr2*^*−/−*^ BMDMs were stimulated with TNC-TNF. After 12 h supernatant was taken and assayed for IL-1β, IL-18 and IL-6. **b** BMDMs were treated for 2 h with TNC-TNF and/or Nec-1s and analysed for TNF RNA expression. **c** BMDMs were treated overnight with TNC-TNF and/or different dilutions of Nec-1s (+ = 1 μM, ++ = 5 μM, +++ = 10 μM) and supernatant was assayed for IL-1β, IL-18 and IL-6. **d** BMDMs of WT, *xiap*^*−/−*^ and *xiap*^*−/−*^*ripk1*^*KD/KD*^ were treated with TNC-TNF for 16 h and showed reduced IL-1β, IL-18 and IL-16 production upon loss of RIPK1 kinase activity. **e** Representative western blot of BMDMs treated for 16 h with TNC-TNF and/or Nec-1s. Data shown are mean ± SEM including *n* = 2–4 biological replicates. Experiments were repeated at least three times independently. Statistical significance was calculated using two-way ANOVA with *p < 0.05, **p < 0.01, ***p < 0.001 and ****p < 0.0001.

Since the RNAseq showed an increased enrichment of inflammation, we screened for cytokine and chemokine production. From this assay, we found several cytokines and chemokines further upregulated in *xiap*^*−/*−^ macrophages compared to wildtype when treated with TNC-TNF for 24 h (Fig. S4A). The key cytokines, IL-1β, IL-18, and IL-6, implicated in symptoms of XLP-2 patients were identified as increased compared to treated wildtype macrophages^29^. Using *xiap*^−*/−*^*tnf*^*−/−*^*, xiap*^*−/−*^*tnfr1*^−*/*−^, and *xiap*^*−/−*^*tnfr2*^*−/*−^ macrophages, we determined IL-1β, IL-18, and IL-6 were dependent on TNF production and required both TNFR1 and TNFR2 present for the production of these cytokines (Fig. 5a).

**Fig. 6:**
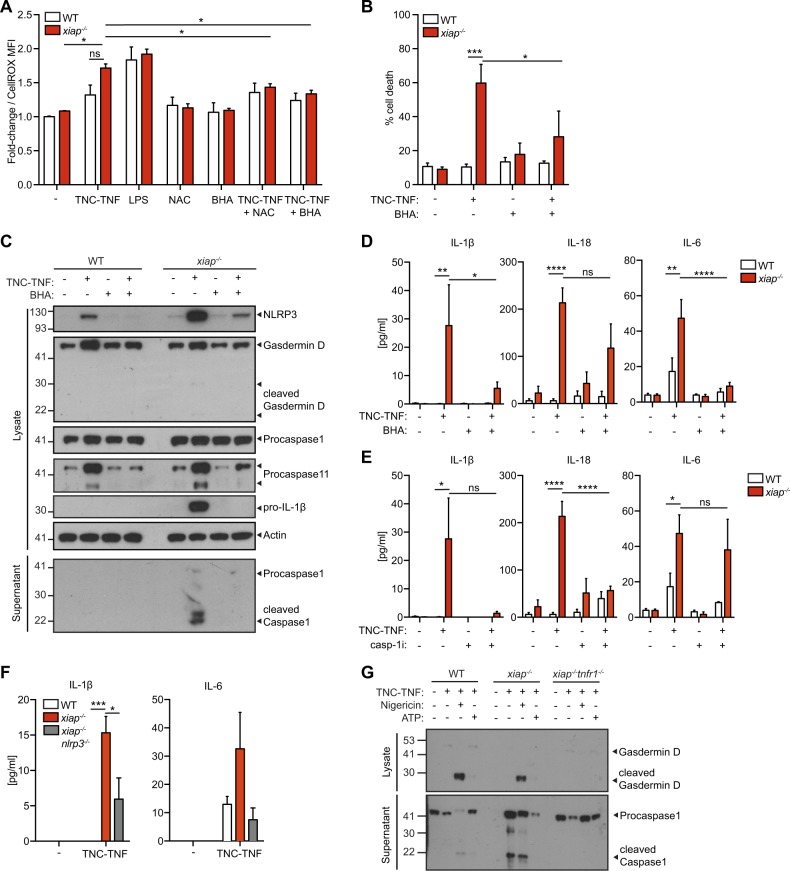
ROS drives NLRP3-inflammasome activation in *xiap*^*−/−*^ macrophages. **a** Cell ROX staining of WT and *xiap*^*−/−*^ macrophages in the presence of TNC-TNF and ROS scavengers NAC (3 mM) and BHA (50 μM). Data are displayed as fold change over WT. **b** BMDMs from WT and *xiap*^*−/−*^ were treated overnight with TNC-TNF and/or BHA. Cell death was assessed by PI uptake on flow cytometry. **c** Cell lysates and supernatant of macrophages treated with TNC-TNF and/or BHA (50 μM) were immunoblotted and probed for gasdermin D, NLRP3, caspase-11, caspase-1 and IL-1β. Western blots are a representative of at least three independent experiments. **d**, **e** BMDMs were treated overnight with TNC-TNF in combination with (**d**) BHA (50 μM) or (**e**) caspase-1 inhibitor (VX-765; 50 μM). IL-1β, IL-18 and IL-6 were measured in the supernatant by multiplex. **f** BMDMs from WT, *xiap*^*−/−*^ and *xiap*^*−/−*^*nlrp3*^*−/−*^ were treated with TNC-TNF and assayed for IL-1β, IL-18 and IL-6 by multiplex. No IL-18 was detected. **g** Cell lysates and supernatant of macrophages treated with TNC-TNF and/or Nigericin (10 μM) or ATP (3 mM) were immunoblotted and probed for caspase-1 and gasdermin D. Western blots are a representative of at least three independent experiments. Data shown are mean ± SEM, including *n* = 3 biological replicates. The experiment was repeated three times independently. Statistical significance was calculated using two-way ANOVA with *p < 0.05, **p < 0.01, ***p < 0.001 and ****p < 0.0001.

Because cell death and inflammation are closely interwined, we asked whether inhibition of RIPK1 kinase activity would also reduce inflammation. The loss of RIPK1 kinase activity resulted in a reduction of TNF mRNA but only in the absence of XIAP (Fig. 5b). Protein levels of IL-1β and IL-6 were easily reduced in response to RIPK1 kinase activity inhibition. However, pre-incubation and high dose of Nec-1s was required to reduce IL-18 (Fig. 5c). Using *xiap*^*−/−*^*ripk1*^*KD/KD*^ macrophages, IL-1β, IL-6, and IL-18 production were completely reduced when treated with TNC-TNF (Fig. 6d). Similar to previous data with LPS stimulation^15,30^, we found RIPK3 loss reduced IL-1β but not IL-6 in *xiap*^*−/−*^*ripk3*^*−/−*^ and *xiap*^*−/−*^*mlkl*^*−/−*^ macrophages in response to TNC-TNF (Fig. S4B). These results imply direct TNFR2 activation in the absence of XIAP leads to increased pro-inflammatory IL-1β, IL-18, and IL-6 in a RIPK1 kinase-dependent manner.

Interestingly, the loss of RIPK1 kinase activity resulted in a slight decrease in gasdermin D and caspase-11 protein levels, as well as cleavage of gasdermin D suggesting the predominant role of RIPK1 kinase activity is to reduce the production of TNF in response to TNFR2 stimulation (Fig. 5e).

### XIAP restricts the activation of NLRP3 inflammasome (signal 2) by regulating ROS production

Previous results suggest that ROS production may influence inflammasome activation. We assayed for ROS production and found in the absence of XIAP, an increased amount of ROS was detected which was reduced by the use of free radical scavengers, BHA or NAC (Fig. 6a). Co-incubation of TNC-TNF and BHA also reduced cell death in the *xiap*^*−/−*^ macrophages (Fig. 6b) and protein levels of NLRP3, gasdermin D and caspase-11 were also reduced and subsequent caspase-1 cleavage was missing (Fig. 6c). ROS scavengers reduced IL-1β and IL-6 but IL-18 levels remained higher than baseline (Fig. 6d).

To determine the significance of caspase-1 and NLRP3 activation in the production of IL-1β and IL-18, we co-incubated cells with caspase-1 inhibitor (VX-765 or Ac YVAD-cmk) with TNC-TNF. The loss of caspase-1 activation resulted in reduced levels of IL-1β, IL-18, and IL-6 in the supernatant of treated *xiap*^*−/−*^ macrophages (Fig. 6e). Using *xiap*^*−/−*^*nlrp3*^*−/−*^ macrophages with *xiap*^*−/−*^ on the Harlin et al. background^31^, we assayed for IL-1β, IL-6, and IL-18 in response to TNC-TNF compared to the matched *xiap*^*−/−*^ strain. Surprisingly, the *xiap*^*−/−*^^31^ did not die in response to TNC-TNF but a decrease in IL-1β was detected and IL-18 was not detected at all (Fig. 6f).

Finally, to determine if TNFR2 activation is sufficient as signal 1, we incubated cells with TNC-TNF and then treated with nigericin or ATP as a second signal. Gasdermin D and caspase-1 cleavage were detected in wildtype macrophages, as well as *xiap*^*−/−*^ macrophages. However, in *xiap*^*−/−*^*tnfr1*^*−/−*^ macrophages, no cleavage was detected (Fig. 6g). Taken together, in the absence of XIAP, increased ROS production leads to NLRP3 induced activation and release of IL-1β.

## Discussion

TNFR2 has been shown to promote survival, differentiation and induce immune suppressive functions^32,33^. However, our data suggest the opposite in the absence of XIAP. Here, we provide evidence that the activation of TNFR2 primes macrophages towards inflammasome activation with dominant expression of proinflammatory cytokines, as well as PRR sensors. Only in the absence of the E3 ligase activity of XIAP do myeloid cells die by pyroptotic activity in response to direct TNFR2 stimulation.

The concept of non-death domain TNF super family receptors (TNFSFR) involved in cell death has been previously reported in cancer cell lines for FN14, CD40, TNFR2, and CD30, acting through a TNF/TNFR1 axis^12,34,35^. Our data differs from recently published results implicating TNFR2 in LPS-induced cell death in the absence of XIAP where necroptosis, pyroptosis and apoptosis is implicated^22^. In the absence of XIAP, TNFR2 induced cell death does not appear to induce caspase activity, nor is there switching from apoptosis to necroptosis as *xiap*^*−/−*^*mlkl*^*−/−*^ and *xiap*^*−/−*^*ripk3*^*−/−*^ in the presence of QVD does not rescue cell death in response to TNFR2 activation. These data suggest that the inflammation may be a separable function to the cell death or preceding cell death activation. Caspase-8 could serve as a scaffold for a NF-kB inducing complex that includes RIPK1 as proposed by Henry et al. to promote inflammation by TRAIL activation^36^. Our data supports this theory as TNFR2 specific activation leads to TNF mRNA production requiring RIPK1 kinase activity, and priming of both pro-inflammatory cytokines and chemokines is present in both wildtype and XIAP deficient cells. These data would suggest RIPK1 kinase activity can mediate inflammation, specifically TNF, in line with previous results linking the kinase activity of RIPK1 to inflammation in the absence of IAPs^23,28,37^. However, further studies would be required to determine if complete inhibition of RIPK1 kinase activity would be achievable in vivo and be effective after inflammation is in progress.

TNFR2-specific activation mediated NOD2 and RIPK2 upregulation, suggesting that TNF may act as a primer for the detection of intracellular pathogens. Indeed, a lack in inflammation has been identified in XLP-2 patients in response to MDP/NOD2 activation through loss of RIPK2 ubiquitylation by XIAP and cIAPs^38–40^. In response to NOD2 stimulation, both mutations in the BIR and RING domain led to a loss in NF-kB^41^. By contrast, our data shows that TNFR2 induced cell death occurs in the absence of E3 ligase activity of XIAP. Whether the loss of E3 ligase activity of XIAP relates to the ROS production is unknown. Further studies in identifying what substrate(s) of XIAP will prevent TNFR2 induced cell death would be beneficial in the treatment of XLP-2 patients.

Our findings are of therapeutic interest as XLP-2 patients have increased inflammasome related cytokines. XIAP deficient patients are characterized with multi-organ inflammation triggered by viral infections, as well as intestinal bowel disease^42,43^. XLP-2 is associated with activated macrophages and lymphocytes and overexpression of pro-inflammatory cytokines including TNF during an HLH episode^29,44^. During viral infections, the increased TNF presence would trigger TNFR1 and TNFR2. Our study provides evidence that inhibition of cell death may not result in reduction of key inflammasome cytokines such as IL-18 in XLP-2 patients and that there is differential regulation of IL-1β in comparison to IL-18 as recently published^45^. Contrary to most situations, targeting TNFR2 with blocking antibodies can be of potential therapeutic interest in XIAP deficient patients to limit inflammation.

## Materials and methods

### Mice

*Xiap*^*−/−*^*, ciap1*^*−/−*^*, ciap2*^*−/−*^, and *ciap1*^*LC*^*ciap2*^*−/−*^ mice were a kind gift from J. Silke from WEHI and were previously described^46,47^. These strains were embryo transferred and maintained in an SPF facility in Zurich. *Tnfr1*^*−/−*^ and *tnfr2*^*−/−*^ mice were a kind gift from A. Fontana and A. Aguzzi, respectively^48^, and were crossed to *xiap*^*−/−*^ mice to generate *xiap*^*−/−*^*tnfr1*^*−/−*^ and *xiap*^*−/−*^*tnfr2*^*−/−*^ mice. *Ripk1*^*K45A/K45A*^ (*ripk1*^*KD/KD*^) mice were a gift from GlaxoSmithKline and were crossed to *xiap*^*−/−*^ mice to generate *xiap*^*−/−*^*ripk1*^*KD/KD*^ mice. All mice used in this study were back-crossed to C57BL/6 mice. All animal experiments were performed at the University of Zurich under the ethical license 186/2015. *Xiap*^*−/−*^ and *xiap*^*−/−*^*nlrp3*^*−/−*^ mice were a kind gift from RA Marsh^31^. *Xiap*^*−/−*^*tnf*^*−/−*^, *xiap*^*−/−*^*ripk3*^*−/−*^, *xiap*^*−/−*^*mlkl*^*−/−*^ and *xiap*^*ΔRING*^ mice were a kind gift from P. Jost and housed at the Technical University of Munich. Experiments were conducted in accordance with GSK policies on the care, welfare, and treatment of laboratory animals.

### Generation of bone marrow derived macrophages and cell lines

To generate bone marrow-derived macrophages (BMDMs), bone marrow was obtained from the tibia and femur of 6–12-week-old mice. Cells were cultured on petri dishes for 5 days in Dulbecco’s modified Eagle medium (DMEM, Gibco) containing 1 g/L glucose, 1% (v/v) penicillin/streptomycin/glutamine (Gibco), 10% FBS (SeraGlobe) and supplemented with 20% (v/v) L929 mouse fibroblast conditioned medium. On day 5, cells were harvested and seeded at 1 × 10^6^ cells/mL into the desired tissue culture plates (e.g., 1 × 10^5^ cells per 96 well). TNFR1-Fas and TNFR2-Fas expressing mouse fibroblasts were obtained from Anja Krippner–Heidenreich^25^ and were cultured in DMEM containing 1 g/L glucose, 1% (v/v) penicillin/streptomycin/glutamine and 10% FBS. HoxB8 progenitor cells were cultured in RPMI 1640 media with 10% (v/v) heat-inactivated FBS, 1% (v/v) penicillin/streptavidin, 7% (v/v) SCF from CHO/SCF conditioned medium, and 0.1 µM 4-hydroxytamoxifen (4-OHT, MedChemExpress)^17^. To differentiate granulocytes from the HoxB8 progenitors, cells were washed twice with PBS and re-suspended at a concentration of 2.5 × 10^4^ cells/mL in media without 4-OHT and differentiated for 5 days.

### Ligands and inhibitors

To produce the agonistic TNFR2 ligands, pCR3 Fc-Flag-TNFR2-specific nonameric murine TNF variant (TNC-TNF), trimeric pCR3 Flag-TNC-TNF or pCR3 Fc-Flag human TNFR1 (TNFR1-TNF), were transfected into 293t cells and purified as previous described^49^. Endotoxin levels were tested and removed (Pierce High Capacity Endotoxin Removal Spin columns, ThermoScientific). TNF variants were used at 100 ng/mL. Inhibitors were used at the following concentrations: Birinapant (500 nM, Chemietek), Compound A (100 nM, Tetralogics), multimeric TNF (mega TNF, 100 ng/mL, Adipogen) Necrostatin-1s (1, 5 or 10 µM, MedChemExpress), Q-VD-OPH (5, 10 or 50 µM, Adipogen), ZVAD-fmk (5, 10 or 50 µM, MedChemExpress), VX-765 (10 µM, Invivogen), BHA (50 µM, Sigma), Nigericin (10 µM, Sigma) and ATP (3 mM, Sigma).

### Antibodies

The following antibodies were used for flow cytometry: CD11b-PeCy7 (clone M1/70, eBioscience), F4/80 (clone BM8, eBioscience), and fixable viability dye (eBioscience). The following antibodies were used for western blotting: phospho-p65, NF-κB2, phospho-ERK, phospho-JNK, phospho-p38, total ERK, total JNK, and total p38 from Cell Signaling Technologies. Total p65 was purchased from Santa Cruz. TRAF2, gasdermin D and caspase-11 were purchased from Abcam, caspase-1 from Adipogen and IL-1β from RnD. cIAP1 was purchased from Human Atlas. RIPK1 and XIAP was purchased from BD Biosciences. Secondary antibodies for western blotting such as donkey anti mouse/rabbit/rat IgG conjugated to HRP are from SouthernBiotec, the donkey anti goat IgG was purchased from Santa Cruz. The neutralizing antibody against TNF (at 200 ng/mL, MP6-XT22) was purchased from BioLegend.

### Cell death analysis

Cells were seeded at a density of 1 × 10^6^ cells/mL. After treatment, cells were trypsinized, washed once and re-suspended in HBSS containing 1 µg/mL propidium iodide (PI) or stained with fixable viability dye (eBioscience). Cells were then assessed for cell death by flow cytometry on a FACS Canto II. Data were analyzed by FlowJo software, version 10.2. Alternatively, cells were directly incubated in the presence of 5 µg/ml propidium iodide and assessed for viability by acquiring both phase contrast and red fluorescence images at 2 h intervals at ×10 magnification over 24 h using the IncuCyte. Confluency and PI fluorescence were measured and analyzed using the IncuCyte Zoom Software (Version 2016A).

### Cell sorting

To isolate primary macrophages from mouse bone marrow, CD11b^+^F4/80^+^ cells were separated using a FACSMelody 3 L machine.

### Caspase activity assay

Cell lysates were treated and lysed in DISC lysis buffer (20 mM Tris-HCl pH 7.5, 150 mM NaCl, 10% (v/v) glycerol, 1% (v/v) Triton X-100, with protease and phosphatase inhibitors) and incubated with 0.5 mM DEVD-AMC. Results were normalized to the protein concentration, that was calculated by BCA (Pierce) according to manufacturer’s instructions.

### Multiplex cytokine analysis

Multiplex cytokine analysis (ProcartaPlex, Thermo Scientific) was performed according to the manufacturer’s instructions and assayed on a Bio-Rad Bioplex machine.

### Western blotting

Supernatant was collected when indicated and precipitated using 4% trichloroacetic acid (TCA) before being pelleted in acetone, boiled and run like lysates described in the following. Cells were lysed using DISC lysis buffer (20 mM Tris-HCl pH 7.5, 150 mM NaCl, 10% (v/v) glycerol, 1% (v/v) Triton X-100, with protease and phosphatase inhibitors). The insoluble fraction of the lysate was pelleted by centrifugation and removed. Lysates were boiled and run on 4–12% Bis-Tris Gel NuPAGE using MOPS buffer (Invitrogen). Proteins were then transferred onto PVDF-membrane (0.2 µm, Thermo Scientific) using the Trans-Blot® Turbo™ Transfer System (Bio Rad) or the Pierce™ Power Blotter (Thermo Scientific), both according to the manufacturer’s instruction. After blocking with PBST containing 5% (w/v) skim milk, membranes were incubated with the indicated primary antibody in either PBST containing 5% skim milk or 5% (w/v) BSA (Fraction V, Sigma) for at least 1 h at RT or overnight at 4 °C. Protein level expression was acquired using WesternBright ECL (Advansta) and Amersham Hyperfilm ECL (GE Healthcare).

### qPCR

RNA was isolated using GENEzol Reagent (Geneaid) according to the manufacturer’s instructions. cDNA was produced using MultiScribe™ Reverse Transcriptase and SYBR Green qPCR master mix (Thermo Fisher Scientific) was used for running the qPCR. Melting curves showed that single products were formed. The following primers were used: TNF (5′: CCA CCA CGC TCT TCT GTC TA; 3′: CAC TTG GTG GTT TGC TAC GA); B2M (5′: TGG TGC TTG TCT CAC TGA CC; 3′ CCG TTC TTC AGC ATT TGG AT). Relative standard curve analysis was performed using the housekeeping gene B2M and unstimulated samples were used as a calibrator for fold-change.

Cells were stimulated with 100 ng/mL of TNC-TNF for 2 h and RNA was extracted using GENEzol Reagent (Geneaid) and cleaned using PureLink RNA Mini Kit (Invitrogen) according to the manufacturer’s instructions. Libraries were prepared and sequenced at the Functional Genomics Center Zurich (Zurich, Switzerland). RNA-Seq data was processed through a standard workflow (https://github.com/csoneson/ARMOR), including read mapping against the mouse reference genome (Ensembl_GRCm38.90) using STAR^50^ and sorting/indexing with samtools^51^. Isoform-level expression estimation using salmon^52^ and gene-level differential expression (DE) analysis was performed using edgeR^53^ with separate contrasts for each genotype (treated with TNC-TNF vs. untreated). Differential expression *t*–statistics used in the geneset analyses were computed using limma-voom^54^.

### Electron microscopy

Cells were fixed in 2.5% glutaraldehyde in 0.1 M cacodylate buffer and scraped from the plate. Transmission electron microscopy was performed by the University of Zurich, ZMB.

### Statistical analysis

All data is presented in mean ± SEM. Figures were prepared in Illustrator CC 2015 (Adobe) and Prism 7 (GraphPad Software). Significance between genotypes and treatments was assessed by Student-*t* test or two-way ANOVA with *p < 0.05, **p < 0.01, ***p < 0.001, ****p < 0.0001 using Prism 7.

## Supplementary information

Supplemental Figure 1

Supplemental Figure 2

Supplemental Figure 3

Supplemental Figure 4

Supplemental Figure 5

Supplemental Figure 6

Supplementary figure legends

Supplemental Dataset 1

Supplemental Dataset 2
