# Correction: TNFR2 induced priming of the inflammasome leads to a RIPK1-dependent cell death in the absence of XIAP

**DOI:** 10.1038/s41419-020-2261-2

**Published:** 2020-01-23

**Authors:** Janin Knop, Lisanne M. Spilgies, Stefanie Rufli, Ramona Reinhart, Lazaros Vasilikos, Monica Yabal, Erika Owsley, Philipp J. Jost, Rebecca A. Marsh, Harald Wajant, Mark D. Robinson, Thomas Kaufmann, W. Wei-Lynn Wong

**Affiliations:** 1 Institute of Experimental Immunology, University of Zürich, Zürich, Switzerland; 2 Institute of Pharmacology, University of Bern, Bern, Switzerland; 3 III. Medizinische Klink, Klinikum rechts der Isar, Technische Universität München, Munich, Germany; 4 UC Department of Pediatrics, Cincinnati Children’s Hospital, Cincinnati, USA; 5 Division of Molecular Internal Medicine, Department of Internal Medicine II, University Hospital Würzburg, Würzburg, Germany; 6 Institute of Molecular Life Sciences and SIB Swiss Institute of Bioinformatics, University of Zürich, Zürich, Switzerland

**Keywords:** Cell death and immune response, Inflammation

**Correction to: Cell Death and Disease**

10.1038/s41419-019-1938-x published online 20 September 2019

The original version of this article contained an error in the name of one of the co-authors (Erika Owsley). This has been corrected in both the PDF and HTML versions of the article.

