# Supplementary material for: TNFR2 induced priming of the inflammasome leads to a RIPK1-dependent cell death in the absence of XIAP
Source: Cell Death Dis. 2019 Sep 20;10(10):700. doi: 10.1038/s41419-019-1938-x (PMC6754467; doi:10.1038/s41419-019-1938-x)
Supplement: Supplementary file 7 — Supplementary figure legends [file 41419_2019_1938_MOESM7_ESM.docx]

**Supplementary Figures**

**Figure S1. Validation of TNFR1 and TNFR2 ligand specificity.** (A) TNFR1-Fas and TNFR2-Fas MF were treated with TR1-TNF overnight. Cell death was measured via PI uptake by flow cytometry. (B) TNFR1-Fas and TNFR2-Fas MF were treated with 80M2 to trimerise human TNFR2, TNC-TNF or trimericFlag-TNC-TNF overnight. (C) WT, *tnfr1^-/-^* and *tnfr2^-/-^* BMDMS were treated with TR1-TNF or TNC-TNF and analysed for NF-kB signaling using western blot. Blots are representative of three independent repeats. (D) Sorted bone marrow macrophages (CD11b+F4/80+) from WT and xiap-/- mice were treated with TNC-TNF overnight. (E) The course of cell death in WT and xiap-/- BMDMs upon TNC-TNF stimulation was monitored over 18h by live imaging using using time-lapse photography (Incucyte). (F,G) WT and *xiap^-/-^* (F) HoxB8 progenitors and (G) HoxB8 granulocytes were treated with TR1-TNF or TNC-TNF over night. Data shown is mean ± SEM, including n=2-4 biological replicates, experiment was repeated independently for at least three times or (F,G) a minimum of three independent repeats, including triplicates for each experiment. Statistical significance was calculated using two-way ANOVA or (F,G) Student t-test with p **=<0.01, ***=<0.001 and ****=<0.0001.

**Figure S2. TNFR2 mediated cell death is due to soluble TNF.** (A) WT and *xiap^-/-^* BMDMs were treated with TNC-TNF or in combination with anti-TNFα for 24h. Supernatant was then transferred onto TNFR1-Fas MF cells and after another 24h cell death was measured via PI uptake by flow cytometry. Data is shown as mean ± SEM, including n=5 biological replicates and three independent experiments have been performed. Statistical significance was calculated using two-way ANOVA with p ****=<0.0001.

**Figure S3. TNFR2-TNF induced cell death in *xiap^-/-^* BMDMs is independent of necroptosis or caspase activity.** (A) BMDMs were treated with TNC-TNF or Compound A (positive control, 100nM) for 12h, cells were lysed and caspase activity was measured over time using a fluorogenic DEVD AMC assay. Caspase activity is represented as fold change over untreated. (B) WT, *xiap^-/-^, xiap^-/-^ripk3*^-/-^, *xiap^-/-^mlkl^-/-^* BMDMs were treated with TNC-TNF and monitored over 18 hours for cell death by PI uptake using time-lapse photograph. Data shown is mean ± SEM including n=3 biological replicates. Experiment was repeated three times independently. Statistical significance was calculated using two-way ANOVA with p *=<0.05, **=<0.01, ***=<0.001, ****=<0.0001.

**Figure S4. Hierarchical clustering of genes differentially expressed in either wildtype, *xiap^-/-^* and *xiap^-/-^tnfr1^-/-^* macrophages in response to TNC-TNF.** BMDMs were treated for 2h with TNC-TNF and transcriptional profiling of the RNA was performed. Normalized expression of genes identified that were differentially regulated with FDR<0.05 were enriched and analyzed for biological function including transcription factors, inflammatory responses and cell death using gene ontology. normalised expression. Colour scale is log(counts-per-million).

**Figure S5: TNFR2 induced cytokine production in *xiap^-/-^* BMDMs is dependent on RIPK1 kinase activity.** WT and *xiap^-/-^* BMDMs were treated with TNC-TNF. (A) After 0h, 8h and 24h of stimulation supernatant was taken and assayed for IL-1β, IL-18, IL-6, CXCL1, CCL11, CCL2, CCL3, and CXCL2 by multiplex. (B) BMDMs of indicated genotypes were treated with TNC-TNF for 12h and supernatant was assayed for IL-1β and IL-6. Data shown is mean ± SEM including n=3 biological replicates. Experiment was repeated three times independently. Statistical significance was calculated using two-way ANOVA with p *=<0.05, ***=<0.001 and ****=<0.0001.

**Figure S6.** **Graphical abstract**. (A) TNFR2 stimulation primes the inflammasome machinery in a RIPK1 dependent manner by upregulating NLRP3, IL-1β and pro-caspase-1. Only in the absence of XIAP does increased ROS production activate the inflammasome, leading to caspase-1 cleavage and gasdermin D mediated pyroptosis with the release of mature IL-1β and IL-18.

**Table S1. RNAseq data of wildtype, *xiap^-/-^* and *xiap^-/-^tnfr1^-/-^* macrophages in response to TNC-TNF.** BMDMs were treated for 2h with TNC-TNF and transcriptional profiling of the RNA was performed.

**Table S2. Gene set enrichment analysis of TNFR2-induced gene signatures.**
